# Randomized, controlled trial comparing laryngeal mask versus endotracheal intubation during neonatal resuscitation---a secondary publication

**DOI:** 10.1186/s12887-016-0553-6

**Published:** 2016-01-25

**Authors:** Chuanzhong Yang, Xiaoyu Zhu, Weibin Lin, Qianshen Zhang, Jinqiong Su, Bingchun Lin, Hongmao Ye, Renjie Yu

**Affiliations:** Neonatal Department, Shenzhen Maternal and Child Healthcare Hospital Affiliated to Southern Medical University, No.2004 Hong Li Road, Futian District, Shenzhen, 518028 China; Neonatal Department, the Third Hospital of Peking University, Beijing, China; Neonatal Department, the First Hospital Affiliated to Tsinghua University, Beijing, China

**Keywords:** Neonate, Resuscitation, Positive pressure ventilation, Laryngeal mask airway, Endotracheal intubation

## Abstract

**Background:**

This study aimed to study the feasibility, efficacy and safety of using laryngeal mask (LM) ventilation compared with endotracheal intubation (ETI) during neonatal resuscitation.

**Methods:**

Neonates with a heart rate below 60 beats per minute despite 30 s of face mask ventilation were assigned quasi-randomly (odd/even birth date) to LM (*n* = 36) or ETI (*n* = 32) ventilation. Differences in first attempt insertion success, insertion time, Apgar score, resuscitation outcome, and adverse effects were compared.

**Results:**

There were no significant differences in first attempt at successful insertion (LM, 94.4 % vs. ETI, 90.6 %), insertion time (LM, 7.58 ± 1.16 s vs. ETI, 7.89 ± 1.52 s), Apgar score at 1 and 5 min, response time, ventilation time, successful resuscitation (LM, 86.1 % vs. ETI,  96.9 %), and adverse events (LM, *n* =3 vs. ETI, *n* =4) between groups.

**Conclusions:**

Laryngeal mask ventilation is an effective alternative to endotracheal intubation during resuscitation of depressed newborns who do not respond to face-mask ventilation. During an emergency, laryngeal mask ventilation may be a preferred technique for medical staff who are unable to acquire or maintain endotracheal intubation skills. Trial registration: Current Controlled Trials ChiCTR-IOQ-15006488. Registered on 2 June 2015.

## Background

The most important intervention in neonatal resuscitation is to achieve effective ventilation and this requires establishing an open airway. An open airway is usually achieved by a face mask or endotracheal tube. However, during neonatal resuscitation, achieving an effective seal with a face mask may be difficult, and providers frequently have difficulty acquiring and maintaining endotracheal intubation (ETI) skills [1–3]. The laryngeal mask (LM) is a supraglottic airway device that is inserted without instruments and covers the glottis with a low pressure seal [4]. Recent international resuscitation guidelines have recommended the LM when bag-mask ventilation (BMV) is ineffective and/or endotracheal intubation (ETI) is unsuccessful or unfeasible [5–9]. In a previous study [10], we showed that the LM and BMV have a similar efficacy and safety during neonatal resuscitation. Advantages to the LM include a higher successful resuscitation rate and decreased total ventilation time. To date, there are limited studies that have far compared the LM with ETI during the resuscitation of severely depressed newborns. In this study, we compared the feasibility, efficacy, and safety of LM ventilation with ETI during neonatal resuscitation.

## Methods

### Setting and patients

**Fig. 1:**
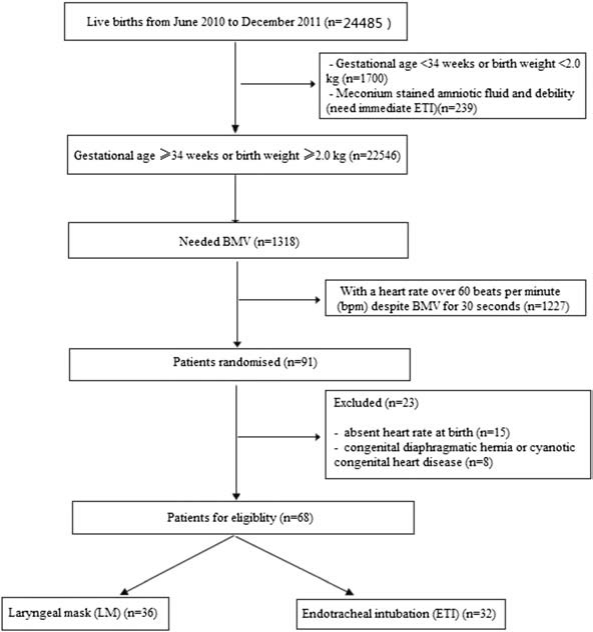
Flow chart of this clinical trial

A prospective, quasi-randomized study that compared LM ventilation with ETI in neonatal resuscitation was conducted from June 2010 to December 2011 at the Maternal and Child Healthcare Hospital of Shenzhen Affiliated to South Medical University (China). The total number of live births was 24,485, which included 9242 born by cesarean section and 15,004 born by vaginal delivery. The number of newborns with a gestational age ≥ 34 weeks or birth weight ≥ 2.0 kg was 22,546, which included 7891 born by cesarean section and 14,655 born by vaginal delivery. Among them, 1318 live births needed BMV (Fig. 1).

Inclusion criteria were a gestational age ≥ 34 weeks, or anticipated birth weight ≥ 2.0 kg, with a heart rate below 60 beats per minute (bpm), despite BMV for 30 s. Exclusion criteria included absent heart rate at birth and known major congenital malformations (e.g., congenital diaphragmatic hernia or cyanotic congenital heart disease).

The study was approved by the institutional ethics committee and informed consent was obtained from parents. For non-emergency delivery, we obtained the informed consent in advance. However, for an emergency delivery, we obtained a post-hoc informed consent.

For BMV, we used a 240-mL resuscitator (Mercury Medical USA) with a size-2 facemask (Ningbo David), and the oxygen concentration to start resuscitation was 21 %. The primary outcome of this clinical trial was to identify any differences in the feasibility, efficacy, and safety between LM ventilation and ETI during neonatal resuscitation. We calculated the power of test according to the literature and our previous clinical studies related in our hospital. We involved nine neonatal specialists from a baby-friendly zone for emergency endotracheal intubation.

### Interventions

Enrolled neonates were quasi-randomized to the LM group (size-1 LMA-Classic™, The Laryngeal Mask Company Limited, UK) or the ETI group based on their date of birth. The LM was used on even dates and the ETI on odd dates. The standard LM insertion technique described by Zhu XY was followed [10]. During resuscitation, the LM or ETI was connected to a self-inflating bag for positive pressure ventilation and 40–60 breaths per minute were provided at a pressure of 25–30 cmH_2_O with 100 % oxygen at a flow rate of 5–8 L/min.

For neonates assigned to the LM group, endotracheal intubation was performed if the heart rate remained less than 60 bpm after 30 s of LM ventilation. The method of resuscitation in the ETI group followed the 2005 Neonatal Resuscitation Program guidelines (American Academy of Pediatrics and American Heart Association) [8]. Assisted ventilation continued until the newborn had a heart rate greater than 100 bpm, spontaneous breathing, pink skin color, and good muscle tone. If meconium-stained amniotic fluid was present and the newborn was not vigorous at birth, tracheal suction through an endotracheal tube was performed before positive pressure ventilation. Then the newborn was resuscitated according to the assigned method.

The data collected during resuscitation included Apgar scores at 1 and 5 min after birth, the time required for device insertion, the number of attempts required for successful device insertion, the number of newborns successfully resuscitated, the time required to achieve successful resuscitation, and the total ventilation time. Successful resuscitation was defined as the newborn establishing spontaneous breathing, heart rate greater than 100 bpm, and good muscle tone. The number of neonates who were assigned to the LM group who required a change to ETI was recorded. We obtained the blood gases and glucose levels of cord blood immediately after birth, and obtained the blood gases and blood glucose levels of peripheral arterial samples 1 h after resuscitation respectively.

### Statistical analysis

Data were analyzed using SPSS 13.0. Normally distributed data are reported as means and standard deviations. Data with a skewed distribution were analyzed after natural logarithmic transformation and are reported as the geometric mean and standard deviations, or median and interquartile range. Independent sample t-tests were used for normally distributed data and non-parametric tests were used for non-normally distributed data and non-parametric variables. The χ^2^ test and exact probability tests were used for categorical data. All outcomes were assessed using the intent-to-treat principle. A *p* ≤ 0.05 was considered a statistically significant difference.

## Results

### Baseline characteristics

Sixty-eight newborns (LM = 36, ETI = 32) were randomized during the study period (Table 1). There were no significant differences in sex (*p* = 0.23), birth weight (*p* = 0.98), gestational age (*p* = 0.24), and mode of delivery (*p* = 0.42) between the intervention groups.

**Table 1:** Demographic characteristics of the patients

	LM	ETI	*p*
	(*n* = 36)	(*n* = 32)	
Male (%)	25 (66.7)	18 (56.3)	0.232
Birth weight, g (mean ± SD/range)	3210 ± 55 (2100 to 4100)	3190 ± 58 (2100 to 4100)	0.976
Gestational age (median/range)	38.2 (33–41.3)	38.6 (33–41.3)	0.240
Vaginal delivery (%)	25 (69.4)	23 (71.9)	0.826

### First attempt at successful insertion

The LM was successfully inserted during the first attempt in 34/36 (94.4 %) newborns. Two attempts were required in one newborn. Insertion was not successful in one newborn who was assigned to the LM group and this newborn was successfully resuscitated using ETI. In the ETI group, the device was successfully inserted during the first attempt in 29/32 (90.6 %) newborns. Three newborns required a second attempt. There was no significant difference in the first attempt at successful insertion between the groups (χ^2^ *=* 0.363*, p =* 0.547).

### Effectiveness of resuscitation

All subjects who were assigned to the LM group survived. In one newborn, the LM could not be inserted and the newborn was resuscitated with ETI. In four additional newborns, the heart rate remained < 60 bpm after 30 s of ventilation with the LM and they were resuscitated using ETI. Two newborns assigned to the LM group survived with hypoxic-ischemic encephalopathy (HIE) and one of them survived with moderate/severe HIE. An endotracheal tube was successfully inserted in all newborns who were assigned to the ETI group. One newborn who was assigned to the ETI group died and two infants survived with moderate/severe HIE. There was no significant difference in the proportion of infants who were successfully resuscitated, the 1- or 5- min Apgar score, the time required for device insertion, the time required to achieve successful resuscitation, or the total ventilation time between groups (Tables 2, 3 and 4). In China, we still use the 1-min Apgar score to diagnose neonatal asphyxia.

**Table 2:** Apgar scores at 1 and 5 min after birth

Apgar scores	1 min		5 min	
	LMA	ETI	LMA	ETI
1	8	9	0	0
2	10	9	0	1
3	13	11	0	0
4	5	3	1	3
5	0	0	1	2
6	0	0	4	3
7	0	0	1	2
8	0	0	4	2
9	0	0	4	5
10	0	0	21	14
Value	*Z* = 0.545		*Z* = 4.769	
*p*	0.909		0.688	

Values are numbers

**Table 3:** Effectiveness and duration of resuscitation

	LMA (*n* = 36)	ETI (*n* = 32)	*p*
Successful resuscitation (%)	31(86.11 %)	31(96.88 %)	0.20
Insertion time(s)	7.58 ± 1.16	7.89 ± 1.52	0.34
Response time (s)	34.06 ± 10.56	41.38 ± 27.19	0.14
Ventilation time(s)	137.19 ± 80.14	171.09 ± 84.28	0.10

**Table 4:** Changes in arterial blood gas values and glucose levels before and after resuscitation

	LMA			ETI			*t* ^a^	*p*
	Before	After	Change	Before	After	Change		
PH	7.17 ± 0.09	7.27 ± 0.95	0.10 ± 0.64	7.11 ± 0.94	7.23 ± 0.74	0.16 ± 0.85	−0.325	0.656
PCO_2_ (mmHg)	48.08 ± 11.24	49.78 ± 13.02	0.73 ± 18.04	48.82 ± 11.66	44.85 ± 14.55	−5.8 ± 20.74	1.19	0.240
PO_2_ (mmHg)	37.63 ± 16.67	80.03 ± 12.40	32.6 ± 23.32	40.42 ± 12.2	84.93 ± 10.39	55.26 ± 36.84	−0.053	0.958
BE (mmol/l)	−6.82 ± 6.30	−5.62 ± 3.67	1.20 ± 6.04	−9.87 ± 4.17	−7.40 ± 5.46	2.47 ± 4.89	−0.751	0.455
HCO_3_^−^(mmol/L)	18.10 ± 6.34	19.36 ± 3.12	1.27 ± 7.26	18.53 ± 3.79	17.78 ± 4.12	−0.75 ± 5.16	1.321	0.191
Glucose(mmol/l)	3.05 ± 0.77	3.77 ± 0.93	0.73 ± 0.52	2.84 ± 0.77	3.75 ± 1.31	0.91 ± 0.77	−0.991	0.325

Values are mean ± SD

^a^Paired difference *t*-test

### Adverse effects

Adverse events in the LM group included vomiting (*n* = 2) and mild abdominal distention (*n* = 1). In the ETI group, adverse events included laryngeal edema (*n* = 1), tracheal bleeding (*n* = 1), and pneumothorax (*n* = 2). There was no significant difference in the incidence of adverse effects between the LM and ETI groups (8.33 % vs 12.5 %, *p* > 0.05).

Three newborns in the LM group were delivered in meconium-stained amniotic fluid (MSAF). The meconium was thought to be “thin” in two newborns and they were ventilated with the LM following oro-pharyngeal suction. In a newborn with “thick” MSAF, suction was performed through an endotracheal tube followed by LM ventilation. All of them were successfully resuscitated without complications. Three neonates in the ETI group were delivered in thick MSAF. All of these neonates were suctioned through the endotracheal tube before ETI ventilation and were successfully resuscitated. One neonate developed pneumothorax. No cases of meconium aspiration syndrome were observed in either group.

## Discussion

Approximately 10 % of newborns require some assistance to begin breathing at birth and <1 % require extensive resuscitation measures [8]. The LM is one option for providing respiratory support. Although the LM was introduced in 1981, and first reported for use during neonatal resuscitation by Paterson in 1994 [11], the LM has not been widely adopted. The International Liaison Committee on Resuscitation, the European Resuscitation Council, the American Academy of Pediatrics and the Australian Resuscitation Council have all included the LM in their recent neonatal resuscitation guidelines because effective ventilation can be achieved quickly during an airway emergency [6, 8, 9, 12, 13]. These international consensus guidelines have focused on the use of an LM as a rescue airway, among newborns weighing >2000 g or delivered at ≥34 weeks’ gestation, if face mask ventilation and ETI are unsuccessful [9]. However, our previous study showed that the LM is more effective than face mask ventilation as the primary airway [10]. The present study was designed to compare the effectiveness of LM and ETI ventilation as the initial choice for a secondary airway during neonatal resuscitation when face mask ventilation is unsuccessful.

In our study, the ventilation time in the LM group appeared to be seemed slightly shorter than that in the ETI group, but this was not significant. This bias produced maybe because ventilation was quickly stopped once the LM was spat out or the neonate started to cry, and there were five neonates who were changed to intubation after 30 s of LM resuscitation. However, ventilation in the ETI group was not stopped until spontaneous breathing recovered completely with extubation of the endotracheal tube. Therefore we could not conclude that ventilation with the LM is more effective than ETI.

Compared with ETI, the potential advantages of using an LM include rapid insertion without requiring laryngoscopy and a higher first attempt success rate, even among novice providers [14]. Successful ETI requires more complex training and evidence suggests that current trainees are not mastering this skill [3]. Resuscitation procedures might be delayed and adverse outcomes might occur if ETI cannot be rapidly implemented (<30 s) or if the proper tube position cannot be achieved. An observational (case–control) study by Zanardo et al. compared resuscitation using either the LM (*n* = 43) or ETI (*n* = 18) [15]. The successful resuscitation rate using the LM was 97 % without adverse outcomes. The incidence of an Apgar score <5 at 5 min, subsequent intensive care unit admission, and respiratory insufficiency requiring mechanical ventilation were significantly lower in the LM group than in the ETI group [15]. In a randomized, controlled trial, Esmail et al. reported similar results in providing effective ventilation, time of resuscitation and success rate during the first attempt with either the LM (*n* = 20) or ETI (*n* = 20) [16]. In their study, the insertion time for the LM was 2.5 s longer than that for ETI (10 vs 7.5 s) . In our study, both devices were quickly inserted and we found no difference in the insertion time (LM vs ETI: 7.58 vs ETI 7.89 s) or the first attempt success rate (LM vs ETI: 86.11 % vs 96.88 %) [10]. In the current study, subjects were newborns with moderate or severe asphyxia. Therefore, the efficacy of the LM was 86.1 %, which is lower than that in our previous study (99 %) [10]. Additionally, the time for ETI we took was short. Therefore, the outcome that the success rate for ETI in our study was lower than in the study by Leone et al., which may have been because of the critical condition of newborns [17].

Adverse events in our study included vomiting (*n* = 2) and mild abdominal distention (*n* = 1). In these cases, the LM may not have achieved a good seal over the glottis and air may have leaked into the esophagus during positive pressure ventilation. Vomiting, cough, and laryngospasm may occur if pharyngo-laryngeal reflexes are present when the LM is inserted [18]. These complications may be less likely during neonatal resuscitation immediately after birth because the newborn is likely to be hypotonic and poorly responsive. When the LM is removed following successful resuscitation, the upper airway should be suctioned to remove secretions that could be aspirated.

Our study showed that the LM had similar effectiveness to ETI when resuscitating moderate/severely depressed newborns. There were no statistically significant differences between these two groups in response time, ventilation time, success rate of resuscitation or the Apgar scores at 1 and 5 min after birth between the two groups. The present study confirms that the LM may be used as the first alternative airway, instead of ETI, to provide positive-pressure ventilation among newborns who do not respond to face mask ventilation. Because the LM insertion technique is easy to teach and requires no airway instrumentation, it may be the preferred device for less experienced health providers. If the newborn does not respond to LM ventilation, ETI may still be required. However, there are insufficient data to evaluate the LM during chest compressions or for the administration of emergency tracheal medications.

Additional questions remain concerning the use of the LM during neonatal resuscitation. The seal provided by the LM leaks at approximately 20–25 cmH_2_O airway pressure [19]. Whether the LM can maintain sufficient airway pressure without excessive leakage when ventilating newborn lungs with low compliance is unknown. Tracheal suction through the LM has not been investigated. Additionally, there is the issue of whether the LM can be used in the setting of a non-vigorous newborn with MSAF. The smallest available LM may not be suitable for premature infants. There are several case reports describing successful resuscitation among very low birth weight infants [20, 21]. The smallest neonate ever reported was 1650 g [22]. Current resuscitation guidelines do not recommend the LM for neonates weighing less than 2000 g. The lower limit of birth weight that should be considered for LM use during resuscitation needs to be determined. There are case reports describing the administration of epinephrine and surfactant through the LM during resuscitation in extremely premature infants [20, 21]. However, the efficacy of intratracheal administration through the LM has not been adequately investigated.

In addition, in this study, we used the size-1 LMA-Classic to perform LM ventilation. However, we did not have sufficient experience for new models of LMA. Currently, we still use the classic LMA. The new models of LMA might be more effective [23].

### Limitations of the study

This was an unblinded study, and neither caregivers nor outcome assessors were masked to treatment allocation. Most of the outcomes, such as Apgar sores and successful resuscitation, were somewhat subjective. Additionally, peak inspiratory pressure and transcutaneous SaO_2_ were not measured.

## Conclusions

Our study shows that the LM may be considered as an effective alternative to ETI during neonatal resuscitation. Because the LM can be quickly inserted without instrumenting the airway, we believe that the LM may be particularly useful in hospitals with less experienced providers where ETI skills may be difficult to acquire and maintain. Because 5 out of 36 (13.8 %) patients who were assigned to the LM group were changed to intubation, the LM may be useful for most neonates, but a person with expertise on intubation must be available in this situation.

## Ethics statement

Ethical approval was provided by the medical ethics committee of Shenzhen Maternal and Child Healthcare Hospital Affiliated to South Medical University, Shenzhen, China. We have written informed consent from the parents of the patients.

## Abbreviations

ETI: endotracheal intubation
HIE: Hypoxic-ischemic encephalopathy
LM: laryngeal mask
MSAF: meconium stained amniotic fluid
